# Predicting the recurrence risk of liver metastasis from colorectal cancer: a study based on preoperative CT intratumoral and peritumoral radiomics features

**DOI:** 10.3389/fonc.2025.1662354

**Published:** 2025-09-23

**Authors:** Dongying Zhang, Peiheng Li, Yong Wei, Mingmei Xue, Fangfang Guo, Chenguang Li

**Affiliations:** Department of Radiology, The First Affiliated Hospital of Xinxiang Medical University, Weihui, China

**Keywords:** computed tomography, radiomics, colorectal neoplasms, liver, neoplasm metastasis

## Abstract

**Objective:**

This study aims to explore the value of predicting the recurrence risk of colorectal cancer liver metastasis (CRLM) based on preoperative CT intratumoral and peritumoral radiomics features.

**Methods:**

This study utilized retrospectively collected preoperative CT data of 201 CRLM patients, comprising 145 cases from the hospital one and 56 cases from an external hospital two. Liver metastases were precisely segmented via manual annotation. Subsequently, the intratumoral region of interest (ROI_Intra_) was isotropically dilated to radii of 2 mm, 4 mm, and 6 mm, resulting in peri-tumoral ROIs (ROI_Peri2mm_, ROI_Peri4mm_ and ROI_Peri6mm_). We established the prediction models based on support vector machine (SVM), random forest (RF), and multilayer perceptron (MLP) algorithms. The area under the subject operating characteristic curve (AUC) was used to evaluate the predictive performance.

**Results:**

Compared with SVM and RF, MLP demonstrated superior predictive performance for estimating the recurrence risk of CRLM patients. The best radiomics signatures for predicting the recurrence risk of CRLM were ROI_Intra+Peri4mm_ model, and the AUCs of the ROI_Intra_ model, ROI_Intra+Peri2mm_ model, ROI_Intra+Peri4mm_ model, and ROI_Intra+Peri6mm_ model constructed by MLP are 0.758 (95% confidence interval (CI), 0.621 - 0.865), 0.815 (95% CI, 0.684 - 0.908), 0.855 (95% CI, 0.731 - 0.936), and 0.825 (95% CI, 0.696 - 0.915), respectively, in the external test set.

**Conclusion:**

Preoperative CT-based radiomics features extracted from intra-tumoral (ROI_Intra_) and peritumoral (ROI_Intra+Peri2mm_, ROI_Intra+Peri4mm_, and ROI_Intra+Peri6mm_) regions can effectively predict recurrence risk in CRLM patients.

## Introduction

Colorectal cancer ranks as the third most prevalent cancer worldwide, accounting for approximately 10% of all cancer cases, and represents the second leading cause of cancer-related mortality globally (1, 2). Approximately 25% of colorectal cancer patients develop liver metastases, for which hepatic resection remains the primary curative treatment (3, 4). Despite advances in surgical techniques and medical oncology that have increased resectability rates, multiple studies have demonstrated that the pooled recurrence rate after liver resection remains alarmingly high at 60-80%, constituting the leading cause of mortality in these patients (5, 6). Precise prediction of recurrence risk is therefore critically important for developing personalized therapeutic strategies and accurate prognostic assessments in patients with colorectal liver metastases (CRLM) (7–9). Clinically, the diagnostic utility of liver biopsy is often constrained by its invasive nature, the potential for procedural complications, and the elevated risk of false-negative results attributable to inadequate tissue sampling or sampling errors. As a routine imaging modality for patients with CRLM, preoperative computed tomography (CT) not only provides detailed anatomical information, including the size, location, and morphology of hepatic lesions, but also captures subtle, indirect indicators of tumor biology (10, 11). These microscale features, imperceptible to the human eye, can be systematically extracted by radiomics through the identification of intratumoral patterns, textural heterogeneities, and spatial relationships within CT images, ultimately transforming them into quantifiable and mineable data for enhanced diagnostic and prognostic insights (12–14). Support vector machine (SVM), random forest (RF), and multilayer perceptron (MLP), as established machine learning algorithms with robust feature extraction capabilities, enable the development of predictive models for CRLM recurrence risk (15–18). By integrating radiomics - based CT feature extraction with SVM, RF, and MLP modeling frameworks, this approach offers a novel, non-invasive solution for personalized risk stratification in CRLM management.

Current radiomics - based studies on preoperative CT imaging for CRLM have predominantly focused on risk prediction (19, 20), chemotherapy response (12, 21), and prognosis (22, 23). However, there remains a critical gap in the development of predictive models for postoperative recurrence risk, which constitutes a major determinant of long-term survival in this patient population. Additionally, the evolution and progression of tumors are affected by the interactions between cells within the tumor and constituents in the peritumoral region (24). Previous research indicates that tumors are composed not only of malignant cells but also stromal components, immune elements and inflammatory elements. These factors induce the stromal remodeling, creating a microenvironment conducive to tumor progression (25). The tumor necrosis factor signaling pathway, which is connected to abnormal blood - vessel formation driven by cancer cells, as well as invasion and metastasis, is related to the features of the peritumoral area (26, 27). In light of this, both the tumor’s intratumoral and peritumoral microenvironments are likely reservoirs of pivotal biological signals and CRLM recurrence indicators.

In this study, we develop and validate a radiomics - based recurrence risk prediction model for CRLM using advanced machine learning algorithms, including SVM, RF, and MLP, applied to preoperative CT imaging. By integrating quantitative imaging features extracted from both intratumoral and peritumoral regions, with the optimal peritumoral area associated with recurrence risk identified, our approach bridges the current gap in postoperative recurrence prediction. Furthermore, by incorporating clinical parameters, we establish a novel, non-invasive prognostic tool to guide postoperative surveillance and personalized treatment strategies, ultimately improving clinical outcomes for CRLM patients.

## Materials and methods

### Patient collective

**Figure 1:**
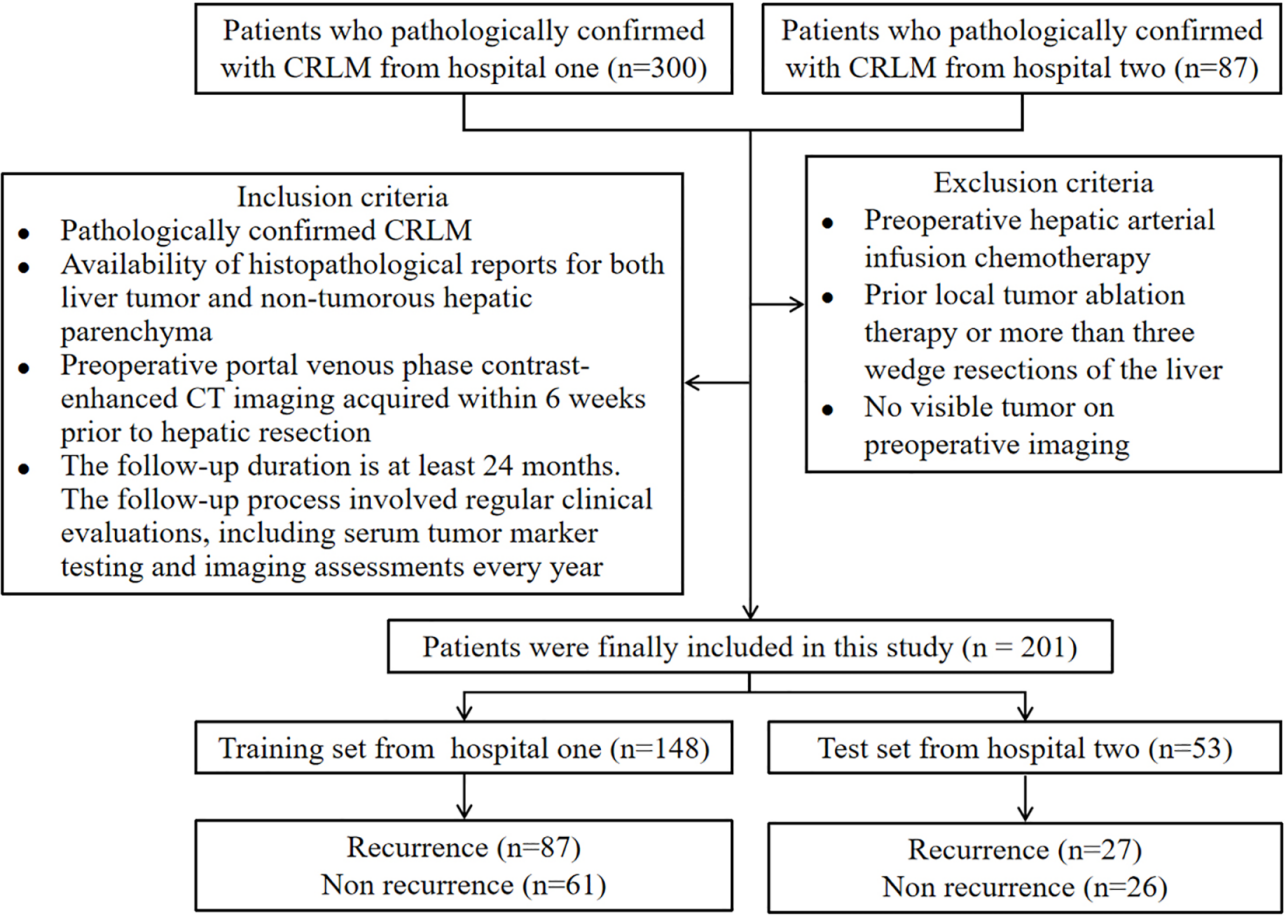
Subject selection flowchart for this experiments.

For this retrospective, multiple-centers, IRB-approved study, 201 patients with proven colorectal cancer and CRLM were identified, including 148 CRLM patients from hospital one and 53 patients from hospital two (**Table 1**). The ethics committee of this institution approved the study and waived informed consent. Inclusion criteria: 1). Pathologically confirmed CRLM; 2). Availability of histopathological reports for both liver tumor and non-tumorous hepatic parenchyma; 3). Preoperative portal venous phase contrast-enhanced CT imaging acquired within 6 weeks prior to hepatic resection; 4). The follow-up duration is at least 24 months. The follow-up process involved regular clinical evaluations, including serum tumor marker testing and imaging assessments every year. Exclusion criteria: 1). Preoperative hepatic arterial infusion chemotherapy; 2). Prior local tumor ablation therapy or more than three wedge resections of the liver; 3). No visible tumor on preoperative imaging. The flowchart of the patient selection process is presented in **Figure 1**. The clinical information available in the datasets included: gender, age, body mass index at operation, carcinoembryonic antigen test, lymph node status, colon primary status, presence of multiple lobes, presence of major comorbidity, maximum tumor size and the liver recurrence status. Recurrence was defined based on a combination of imaging and pathological evidence. Detection of new lesions on cross-sectional imaging with typical radiological features of metastatic disease, e.g., enhancing hepatic nodules, extrahepatic masses, that were not present at baseline and persisted or enlarged on subsequent scans. Histopathological verification of malignant cells from biopsy or surgical resection of suspected recurrent lesions, which served as the gold standard when available.

**Table 1:** Clinical characteristics of CRLM patients in the training and test sets.

Variable	Training set (hospital one)		Test set (hospital two)		z/t/χ^2^ value	*P* value
	Recurrence (n = 87)	Non-recurrence (n = 61)	Recurrence (n = 27)	Non-recurrence (n = 26)		
Age (years)	58.37 ± 12.31	61.11 ± 11.66	63.18 ± 10.58	64.58 ± 12.14	1.360	0.176
Gender					2.581	0.1082
Male	44 (50.57 %)	39 (63.93 %)	19 (70.37 %)	16 (61.54 %)		
Female	43 (49.43 %)	22 (36.07 %)	8 (29.63 %)	10 (38.46 %)		
Body mass index (kg/ m^2^)	26.86 ± 4.86	27.83 ± 5.25	26.56 ± 4.10	23.05 ± 2.85	-1.134	0.257
Carcinoembryonic antigen test	31.79 ± 116.83	36.57 ± 124.98	36.52 ± 58.95	56.55 ± 149.21	-1.385	0.166
Positive lymph node of primary tumor					0.019	0.8903
Yes	29 (33.33 %)	29 (33.33 %)	15 (55.56 %)	12 (46.15 %)		
No	58 (66.67 %)	58 (66.67 %)	12 (44.44 %)	14 (53.85 %)		
Colon primary at operation					0.361	0.5482
Yes	47 (54.02 %)	21 (34.43 %)	9 (33.33 %)	4 (15.38 %)		
No	40 (45.98 %)	40 (65.57 %)	18 (66.67 %)	22 (84.62 %)		
Disease in multiple lobes					4.892	0.0270
Yes	46 (52.87 %)	21 (34.43 %)	10 (37.04 %)	10 (38.46 %)		
No	41 (47.13 %)	40 (65.57 %)	17 (62.96 %)	16 (61.54 %)		
Major comorbidity					3.265	0.0708
Yes	44 (50.57 %)	40 (65.57 %)	15 (55.56 %)	18 (69.23 %)		
No	43 (49.43 %)	21 (34.43 %)	12 (44.44 %)	8 (30.77 %)		
Max tumor size (cm)	3.64 ± 2.22	2.95 ± 2.39	5.08 ± 3.91	4.50 ± 1.66	-2.434	0.015

### Imaging protocols

All study participants underwent a standardized contrast-enhanced CT scan according to predefined imaging protocols. Abdominal imaging was performed using a multidetector CT scanner (Lightspeed 16 and VCT; GE Healthcare, Madison, WI, USA) with the following key settings: autoMA ranged from 220 to 380, a noise index of 12 to 14, a rotation time of 0.7 to 0.8 milliseconds, and a scan delay of 80 seconds (28).

### Intratumoral segmentation and peritumoral dilation

Intratumoral segmentation was performed on CT images using the ITK-SNAP software (Version 3.60, http://www.itk-snap.org). Two radiologists with 8 and 10 years of experience in abdominal imaging diagnosis, respectively, independently carried out the segmentation process. If the region of interest (ROI) defined by two radiologists showed a discrepancy ≥5%, a senior radiologist with two decades of expertise conducted re-segmentation to finalize the ROIs. The segmented ROI served as the ROI_Intra_, which was expanded by 2 mm, 4 mm, and 6 mm into the peritumoral region using standard morphological operations, yielding ROI_Peri2mm_, ROI_Peri4mm_ and ROI_Peri6mm_, respectively. The ROI_Peri k mm_ excluded skin, air, and muscles.

### Data preprocessing

Prior to the extraction of radiomics features, all CT images underwent resampling to achieve a uniform voxel resolution of 1×1×1 mm³. Subsequently, the intensity histograms of the images were discretized using a bin width of 25, ensuring consistent and standardized feature extraction across the dataset. This preprocessing step is crucial for maintaining the comparability and reliability of the extracted radiomics features.

### Radiomics feature extraction

Radiomics features were extracted from the ROI using the open-source software PyRadiomics (Version 2.20, https://github.com/Radiomics/pyradiomics). A comprehensive set of 12 filters, including Original, AdditiveGaussianNoise, Binomial, Normalize, LaplacianSharpening, CurvatureFlow, wavelet, ShotNoise, BoxMean, LoG, DiscreteGaussian, and BoxSigmaImage, were applied to enhance the extraction of radiomics features. A total of 201 patients in this study yielded 1197 high-dimensional radiomics features. To reduce the complexity or bias inherent in the radiomics feature set, dimensionality reduction techniques were employed. The primary objective of feature dimensionality reduction is to simplify the feature space while retaining the most informative features. In the training dataset, we first performed feature dimensionality reduction using the least absolute shrinkage and selection operator (LASSO) regression analysis, with the regularization parameter α set to 0.001 (29). Subsequently, the top 15 features with the highest correlation were selected based on the maximum relevance and minimum redundancy (mRMR) method (30). These methods help in selecting the most discriminative features, thereby improving the performance of subsequent predictive models.

### Model construction

**Figure 2:**
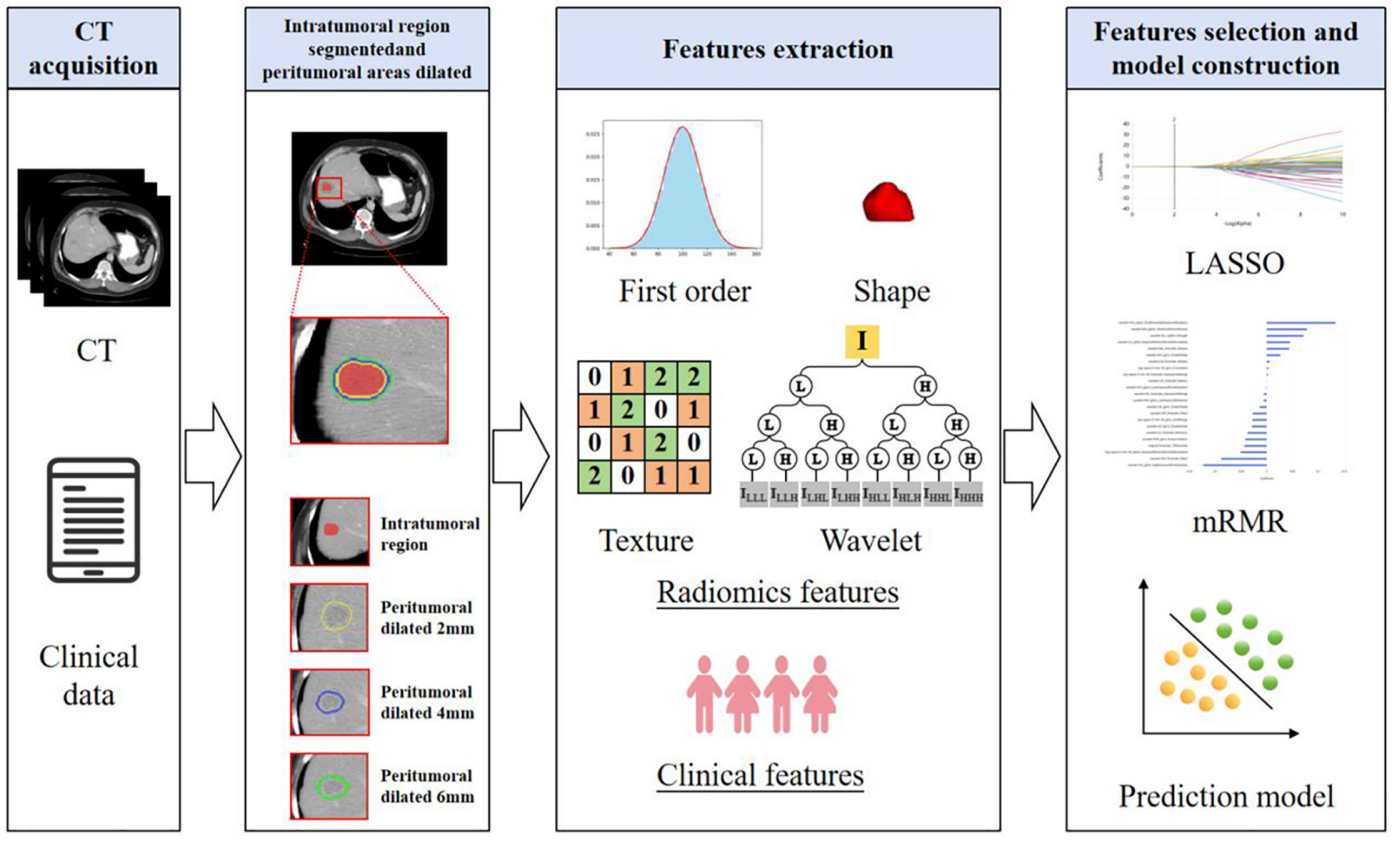
Radiomics analysis and machine learning workflow for predicting the recurrence risk in CRLM patients.

Using radiomics features extracted from ROI_Intra_ and its peritumoral extensions (ROI_Intra+Peri2mm_, ROI_Intra+Peri4mm_, and ROI_Intra+Peri6mm_), predictive models were developed using machine learning algorithms, including SVM, RF, and MLP, to predict recurrence risk in CRLM. The radiomics workflow is shown in **Figure 2**. The details of these classifiers were shown in **Table 2**.

**Table 2:** The details of different classifiers.

Classifier	Parameters
SVM	Penalty factor C: 1.0; Gamma:0.001; Kernel: rbf; Threshold:0.5
RF	Criterion method: gini; Maximum depth of tree: 3; Minimum number of tree leaf: 1; Minimum number of splitter sample: 2; Number of estimators: 200; Threshold: 0.5
MLP	Number of hidden layer: 2, Sizes of hidden layer: 64, Activation: sigmoid, 64, Learning rate: 0.001, Optimizer: Adam optimizer with default parameters (β_1_ = 0.9, β_2_ = 0.999), Regularization: L2, Batch size: 32, Epochs: 200, Initialization: Glorot uniform, Loss function: binary cross entropy

SVM, support vector machine; RF, random forest; MLP, multilayer perceptron

### Statistical analysis

In the analysis of count data, comparisons between groups were performed using the Chi-square test, which is appropriate for categorical variables. For continuous data, comparisons between groups were conducted using either the Mann-Whitney U test or the independent samples t-test, depending on the normality assumptions of the data. The performance of the predictive models was evaluated using several metrics, including the area under the subject operating characteristic curve (AUC), sensitivity, and specificity. Decision curve analysis (DCA) and calibration curve were employed to independently evaluate the stability and clinical net benefit of the predictive model, respectively. These metrics provide a comprehensive assessment of the model’s ability to distinguish between different outcomes. All statistical analyses were conducted using the R software (Version 4.3.3). A significance level of P < 0.05 was adopted to indicate statistically significant differences.

## Results

### Statistical analysis of clinical features

A total of 114 patients with recurrent CRLM and 87 patients without recurrence were included. Among the clinical characteristics, significant differences were observed between the two groups in terms of presence of multiple lobes and the maximum tumor diameter. Consequently, presence of multiple lobes and the maximum tumor diameter were incorporated as clinical indicators into the predictive model (**Table 1**). The two variables were selected due to their statistical significance, suggesting their potential importance in influencing the recurrence risk of CRLM. Including these clinical indicators enhances the model’s ability to accurately predict recurrence by accounting for relevant patient-specific factors.

### Radiomics feature selection and predictive performance

From the CT-based ROIs (ROI_Intra_, ROI_Intra+Peri2mm_, ROI_Intra+Peri4mm_, and ROI_Intra+Peri6mm_), 15 radiomics features were ultimately selected based on their significant association with recurrence risk. The extracted radiomics features by ROI_Intra_, ROI_Intra+Peri2mm_, ROI_Intra+Peri4mm_, and ROI_Intra+Peri6mm_, which are presented in **Tables 3**, **4**, **5**, and **6**. The results of recurrence risk prediction for CRLM patients, achieved by integrating radiomics features with clinical characteristics and constructing predictive models using SVM, RF, and MLP, are presented in **Table 3**, **4**. The predictive performance of the three machine learning models (SVM, RF, and MLP) improved consistently when incorporating peritumoral regions into the radiomics signature. The optimal performance was observed with the MLP model using ROI_Intra+Peri4mm_, achieving the highest AUC of 0.905 (95% CI: 0.846 - 0.947) in the training set and 0.855 (95% CI: 0.731 - 0.936) in the test set. The significance level *P* for all nine models was less than 0.0001 (**Table 7**).

**Table 3:** The key radiomics features selected out through ROI_Intra_.

Features type	Radiomics features	Correlation coefficient
Texture feature	original_glrlm_LongRunHighGrayLevelEmphasis	2.616
First order statistic	original_firstorder_Mean	2.455
Shape feature	original_shape_Sphericity	1.979
Texture feature	log-sigma-3-0-mm-3D_glcm_Id	1.718
Texture feature	wavelet-HLL_glrlm_LowGrayLevelRunEmphasis	1.627
Shape feature	original_shape_LeastAxisLength	1.406
Texture feature	wavelet-HHL_glrlm_LowGrayLevelRunEmphasis	1.072
Texture feature	original_glszm_SmallAreaLowGrayLevelEmphasis	0.962
Texture feature	wavelet-LHH_ngtdm_Coarseness	0.874
First order statistic	wavelet-LHL_firstorder_Energy	0.691
Texture feature	wavelet-HLH_glcm_MaximumProbability	0.439
Texture feature	wavelet-LLH_glcm_Contrast	0.323
Texture feature	wavelet-HHH_glrlm_LongRunLowGrayLevelEmphasis	0.254
Texture feature	wavelet-HHL_glcm_Contrast	0.108
Texture feature	log-sigma-5-mm-3D_glszm_SizeZoneNonUniformity	0.057

**Table 4:** The key radiomics features selected out through ROI_Intra+Peri2mm_.

Features type	Radiomics features	Correlation coefficient
Texture feature	wavelet-LLH_glcm_Correlation	3.029
Texture feature	wavelet-LLH_glcm_DifferenceEntropy	3.006
Texture feature	wavelet-LLH_gldm_DependenceNonUniformity	2.861
Texture feature	wavelet-HLH_glcm_ClusterTendency	2.554
Texture feature	wavelet-HHL_glszm_GrayLevelNonUniformity	2.175
Texture feature	log-sigma-5-mm-3D_glcm_MCC	1.401
Texture feature	original_glcm_SumAverage	1.272
Shape feature	original_shape_SphericalDisproportion	1.143
Texture feature	wavelet-LLH_glszm_HighGrayLevelZoneEmphasis	1.015
Texture feature	log-sigma-3-mm-3D_glrlm_ShortRunHighGrayLevelEmphasis	0.823
Shape feature	original_shape_LeastAxisLength	0.753
Texture feature	wavelet-LLL_gldm_GrayLevelVariance	0.554
Texture feature	wavelet-LLL_gldm_LowGrayLevelEmphasis	0.371
Shape feature	original_shape_SurfaceArea	0.118
Shape feature	original_shape_Maximum3DDiameter	0.082

**Table 5:** The key radiomics features selected out through ROI_Intra+Peri4mm_.

Features type	Radiomics features	Correlation coefficient
Texture feature	wavelet-HHL_glszm_HighGrayLevelZoneEmphasis	3.917
Texture feature	wavelet-HHL_glszm_LargeAreaEmphasis	3.558
Texture feature	wavelet-HHL_glszm_ZoneEntropy	2.614
Texture feature	wavelet-HHL_ngtdm_Coarseness	2.305
Texture feature	log-sigma-3-mm-3D_ngtdm_Coarseness	1.923
Texture feature	original_gldm_GrayLevelNonUniformity	1.824
Texture feature	wavelet-LLL_ngtdm_Busyness	1.438
First order statistic	log-sigma-5-mm-3D_firstorder_TotalEnergy	1.117
Texture feature	original_gldm_LargeDependenceHighGrayLevelEmphasis	0.925
Texture feature	wavelet-LLL_glszm_ZonePercentage	0.759
Texture feature	wavelet-LLL_glszm_LargeAreaEmphasis	0.668
First order statistic	log-sigma-5-mm-3D_firstorder_Median	0.503
Texture feature	original_gldm_SmallDependenceHighGrayLevelEmphasis	0.249
Texture feature	wavelet-LLL_glrlm_ShortRunEmphasis	0.176
Texture feature	wavelet-LLL_glszm_GrayLevelNonUniformityNormalized	0.094

**Table 6:** The key radiomics features selected out through ROI_Intra+Peri6mm_.

Features type	Radiomics features	Correlation coefficient
Texture feature	wavelet-LLL_glrlm_GrayLevelNonUniformityNormalized	2.734
Texture feature	wavelet-LLL_glrlm_LongRunEmphasis	2.439
Texture feature	log-sigma-5-mm-3D_glcm_JointEntropy	2.402
First order statistic	original_firstorder_RobustMeanAbsoluteDeviation	2.078
First order statistic	wavelet-HLH_firstorder_TotalEnergy	1.891
Texture feature	wavelet-LLL_glszm_GrayLevelNonUniformity	1.547
Texture feature	log-sigma-5-mm-3D_gldm_LowGrayLevelEmphasis	1.430
First order statistic	original_firstorder_Mean	1.271
Texture feature	wavelet-LLL_glszm_SizeZoneNonUniformityNormalized	0.869
Texture feature	log-sigma-5-mm-3D_gldm_GrayLevelNonUniformity	0.724
Texture feature	wavelet-HLH_glcm_ClusterProminence	0.502
Texture feature	wavelet-LLL_glszm_ZonePercentage	0.418
Shape feature	original_shape_Maximum2DDiameterColumn	0.401
Texture feature	wavelet-HLH_glcm_Contrast	0.259
First order statistic	original_firstorder_90Percentile	0.127

**Table 7:** Performance metrics SVM, RF, and MLP models across different ROI radiomics signature in the training set.

Radiomics signature	Algorithm	AUC (95% CI)	Sensitivity (%)	Specifcity (%)	Standard error
ROI_Intra_	SVM	0.844 (0.776 - 0.899)	86.21	86.89	0.0346
	RF	0.860 (0.793 - 0.911)	87.36	88.52	0.0343
	MLP	0.872 (0.807 - 0.921)	89.66	90.16	0.0344
ROI_Intra+Peri2mm_	SVM	0.861 (0.795 - 0.912)	87.36	88.52	0.0328
	RF	0.898 (0.837 - 0.941)	89.66	90.16	0.0317
	MLP	0.905 (0.846 - 0.947)	90.80	90.16	0.0284
ROI_Intra+Peri4mm_	SVM	0.893 (0.832 - 0.938)	88.51	91.80	0.0302
	RF	0.909 (0.850 - 0.950)	90.80	90.16	0.0269
	MLP	0.929 (0.875 - 0.965)	94.25	93.44	0.0247
ROI_Intra+Peri6mm_	SVM	0.906 (0.847 - 0.948)	91.95	90.16	0.0285
	RF	0.911 (0.854 - 0.952)	88.51	90.16	0.0260
	MLP	0.914 (0.856 - 0.954)	91.95	86.89	0.0259

95% CI, confidence interval; SVM, support vector machine; RF, random forest; MLP, multilayer perceptron

**Figure 3:**
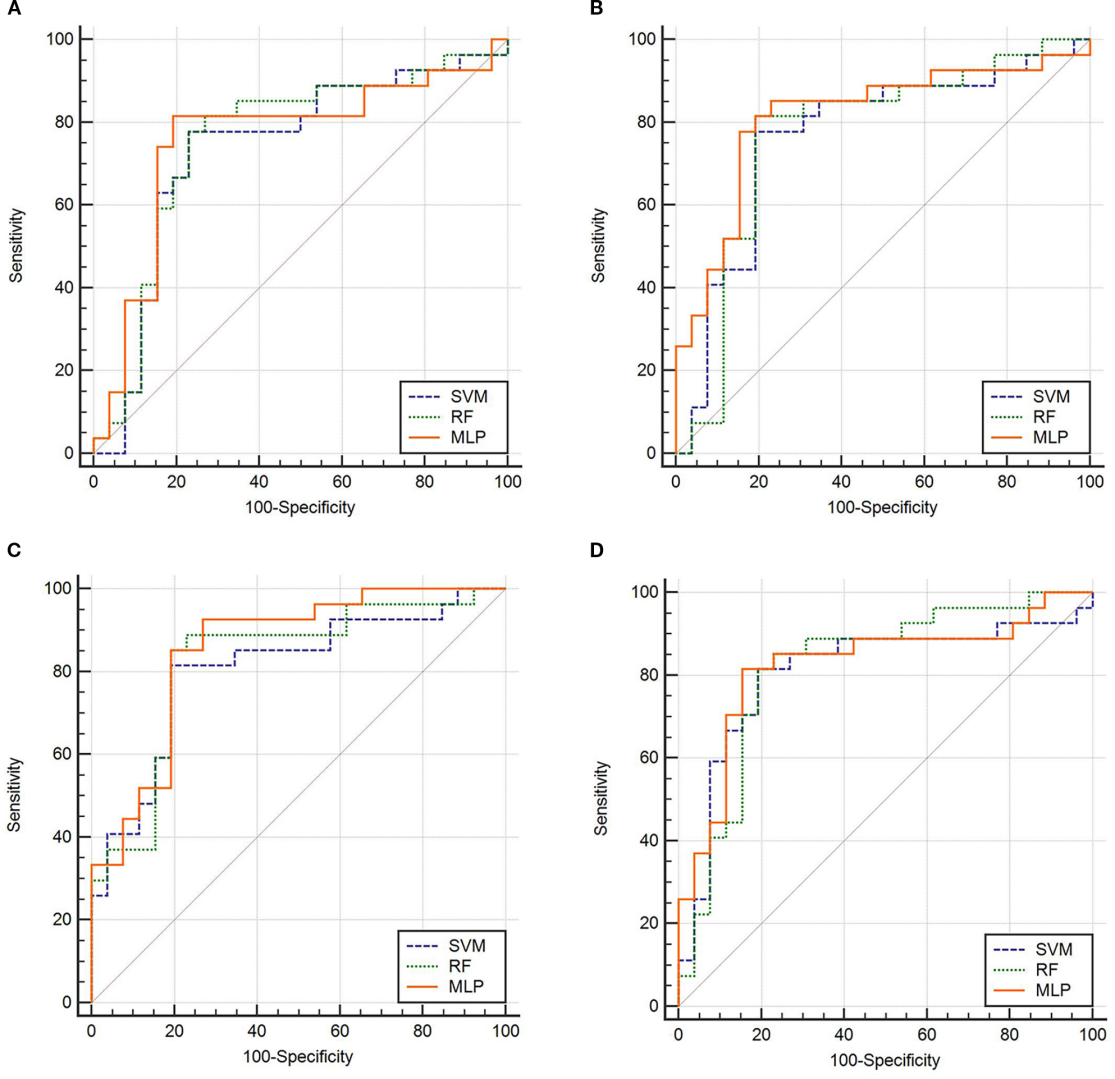
ROC of the predictive models constructed by different radiomics signatures in the test set. **(A)** ROI_Intra_, **(B)** ROI_Intra+Peri2mm_, **(C)** ROI_Intra+Peri4mm_, **(D)** ROI_Intra+Peri6mm_.

**Figure 4:**
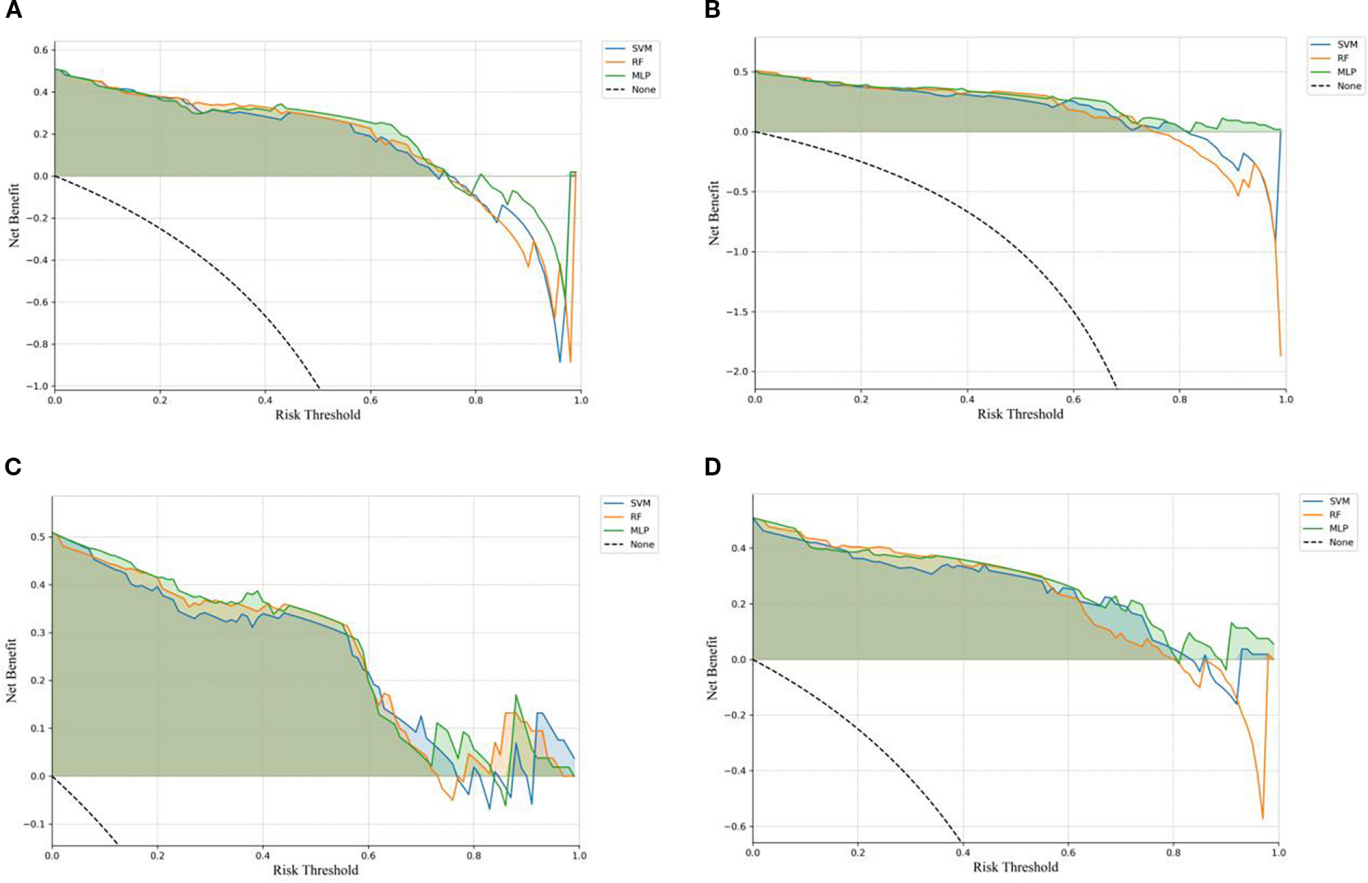
DCA of the predictive models constructed by different radiomics signatures in the test set. **(A)** ROI_Intra_, **(B)** ROI_Intra+Peri2mm_, **(C)** ROI_Intra+Peri4mm_, **(D)** ROI_Intra+Peri6mm_.

**Figure 5:**
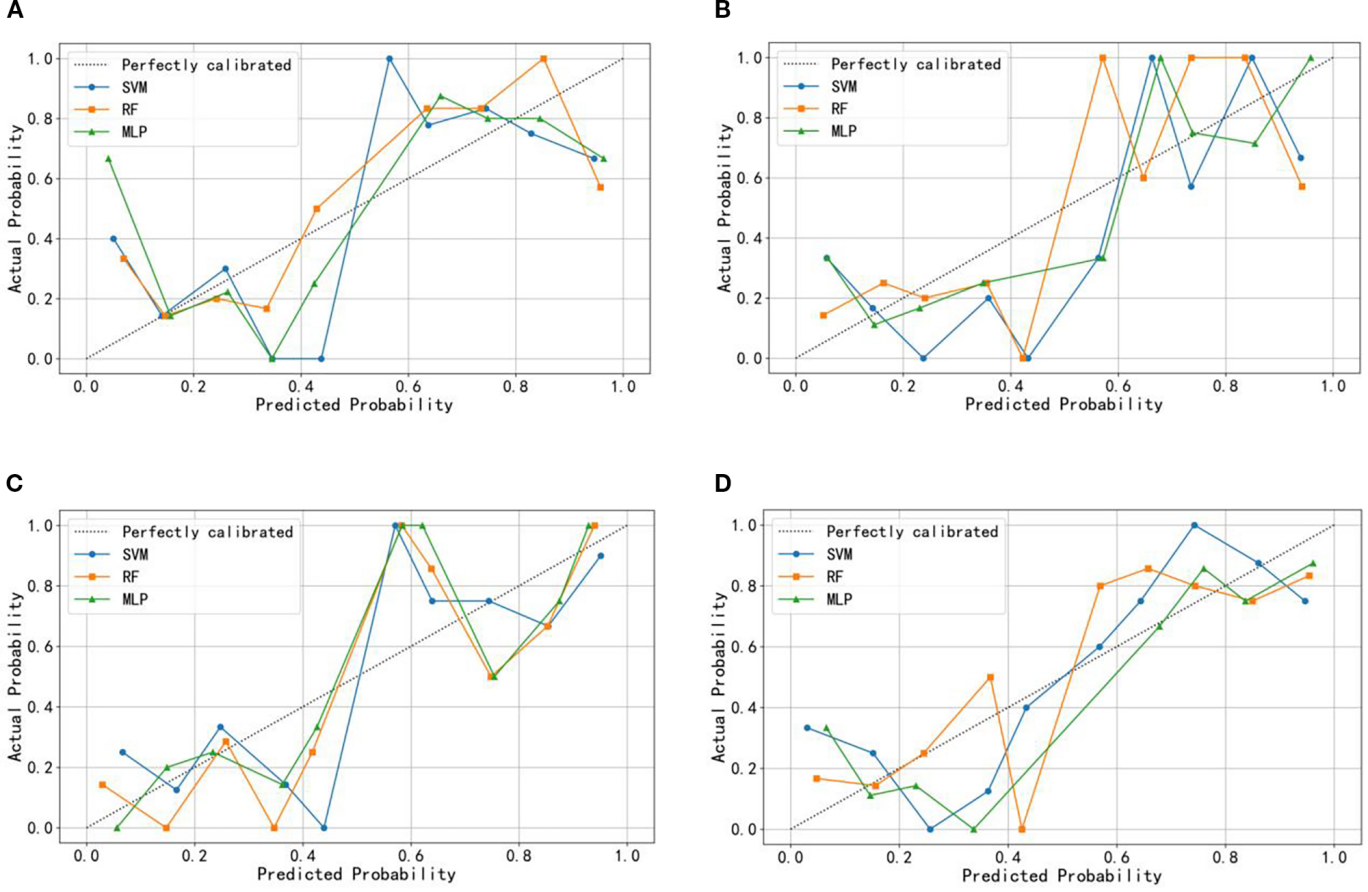
Calibration curve of the predictive models constructed by different radiomics signatures in the test set. **(A)** ROI_Intra_, **(B)** ROI_Intra+Peri2mm_, **(C)** ROI_Intra+Peri4mm_, **(D)** ROI_Intra+Peri6mm_.

The ROC curves (**Figure 3**) illustrate that models constructed using CT intratumoral and peritumoral radiomics features are effective in predicting the recurrence risk of CRLM, with all models achieving AUC values exceeding 0.735 in the test set. Based on the H-L test, the *P*-values for the ROI_Intra_ model, ROI_Intra+Peri2mm_ model, ROI_Intra+Peri4mm_ model, and ROI_Intra+Peri6mm_ model by SVM compared to actual observations were 0.2262, 0.2298, 0.3631, and 0.0110, respectively. The *P*-values for the ROI_Intra_ model, ROI_Intra+Peri2mm_ model, ROI_Intra+Peri4mm_ model, and ROI_Intra+Peri6mm_ model by RF compared to actual observations were 0.4532, 0.3038, 0.3190, and 0.1586, respectively. The *P*-values for the ROI_Intra_ model, ROI_Intra+Peri2mm_ model, ROI_Intra+Peri4mm_ model, and ROI_Intra+Peri6mm_ model by MLP compared to actual observations were 0.3068, 0.3845, 0.8556, and 0.4218, respectively. The decision curve analysis evaluated the clinical utility of all the predictive models (ROI_Intra_, ROI_Intra+Peri2mm_, ROI_Intra+Peri4mm_, and ROI_Intra+Peri6mm_) across a range of threshold probabilities (**Figure 4**). The calibration curves assessed the agreement between predicted probabilities and observed outcomes (**Figure 5**). The results demonstrated that all models provided net benefit, indicating their potential clinical applicability for predicting recurrence risk in CRLM patients (**Table 8**).

**Table 8:** Performance metrics SVM, RF, and MLP models across different ROI radiomics signature in the test set.

Radiomics signature	Algorithm	AUC (95% CI)	Sensitivity (%)	Specifcity (%)	Standard error
ROI_Intra_	SVM	0.735 (0.596 - 0.847)	77.78	76.92	0.0744
	RF	0.755 (0.617 - 0.863)	77.78	76.92	0.0724
	MLP	0.758 (0.621 - 0.865)	81.48	80.77	0.0727
ROI_Intra+Peri2mm_	SVM	0.766 (0.630 - 0.872)	77.78	80.77	0.0703
	RF	0.771 (0.635 - 0.875)	81.48	80.77	0.0712
	MLP	0.815 (0.684 - 0.908)	77.78	84.62	0.0629
ROI_Intra+Peri4mm_	SVM	0.806 (0.675 - 0.902)	81.48	80.77	0.0620
	RF	0.825 (0.696 - 0.915)	85.19	80.77	0.0602
	MLP	0.855 (0.731 - 0.936)	85.19	80.77	0.0529
ROI_Intra+Peri6mm_	SVM	0.808 (0.676 - 0.903)	81.48	80.77	0.0661
	RF	0.821 (0.691 - 0.912)	81.48	80.77	0.0615
	MLP	0.825 (0.696 - 0.915)	81.48	84.62	0.0615

95% CI, confidence interval; SVM, support vector machine; RF, random forest; MLP, multilayer perceptron

## Discussion

Radiomics - based analyses of preoperative CT imaging have been extensively validated for predicting metastatic risk, chemotherapy response, and prognosis in CRLM patients. For example, a study by Jing et al. showed that a radiomics model based on CT for preoperative prediction of liver metastases after surgery for colorectal cancer, with AUC of 0.761 in the test set (19). Karagkounis et al. constructed the radiomics prediction model using CT features of 85 Patients who underwent resection for CRLM, and the results showed that this model can provide a valuable reference for pathological response assessment (13). However, the potential of radiomics for recurrence risk stratification remains relatively underexplored in this patient population. This knowledge gap is particularly concerning given that recurrence patterns in CRLM are highly heterogeneous, with liver metastases and local hepatic recurrences exhibiting distinct clinical behaviors. Meanwhile, existing studies predominantly focus on intralesional features, overlooking the recurrence value of peritumoral texture patterns that may reflect microenvironmental changes associated with tumor dissemination. To address these gaps, our study systematically evaluated the predictive performance of radiomics models for recurrence risk by constructing separate SVM, RF, and MLP algorithms using four distinct radiomics feature sets derived from the intra-tumoral ROI (ROI_Intra_) and its peritumoral extensions (ROI_Intra+Peri2mm_, ROI_Intra+Peri4mm_, and ROI_Intra+Peri6mm_).

Biologically, the peritumoral region plays a critical role in tumor progression, as it is where cancer cells interact with stromal tissue, immune cells, and blood vessels, processes that drive local invasion and recurrence (13). Studies on hepatic metastases have shown that pathological changes extend beyond the visible tumor boundary, with distinct biological signatures observed at varying distances from the tumor edge (31). Specifically, the 2mm zone primarily reflects immediate tumor-stroma interactions, including early invasive activity and extracellular matrix remodeling. The 4mm zone captures broader paracrine effects and immune responses that mediate tumor survival and spread. The 6mm zone encompasses more distant microenvironmental changes, such as hepatic sinusoidal remodeling, which can facilitate micrometastasis formation. For instance, Shang et al. predicted the invasiveness of lung adenocarcinoma by analyzing radiomic features extracted from both tumor cores and 4mm peritumoral regions on CT imaging (32). Qin et al. established through MRI analysis that systematic evaluation of peritumoral ROIs expanded by 2mm, 4mm, and 6mm beyond tumor margins provides clinically significant predictive value for assessing pathological treatment response in locally advanced rectal cancer patients following neoadjuvant chemoradiotherapy (33). Clinically, these distances align with previous radiomic studies on malignancies, where 2 - 6mm peritumoral regions have been associated with recurrence risk and treatment response. We selected 2mm, 4mm, and 6mm to span this clinically relevant range, allowing us to capture both proximal and distal microenvironmental influences on recurrence.

The extraction of radiomics features is based on the principles of quantitative imaging analysis, which systematically quantifies the spatial and intensity distributions of voxel patterns within medical images. These features capture tumor heterogeneity and microenvironmental characteristics, thereby possessing significant biological relevance in medical research. As shown in **Table 3**, the first column displays the categories corresponding to the radiomics features, the second column lists the names of the selected features, and the third column presents the mRMR correlation coefficients between the radiomics features and the recurrence risk. We have analyzed the biological significance of the 3 radiomics features that are most strongly associated with the risk of recurrence. Original_glrlm_LongRunHighGrayLevelEmphasis measures the distribution of long contiguous pixel runs with high gray-level intensity values. The high value may correspond to highly ordered proliferative regions within the tumor or high cell density zones around necrotic areas, and is associated with the degree of tumor differentiation. Original_firstorder_Mean represents arithmetic mean of all pixel intensities within the tumor ROI. Low mean values may indicate necrotic areas, while high values suggest vascularized tumor regions. Original_shape_Sphericity measures how closely the tumor shape approximates a perfect sphere. Lower sphericity correlates with infiltrative growth patterns and desmoplastic reaction.

The radiomics features extracted by ROI_Intra+Peri2mm_ are shown in **Table 4**. The top 3 radiomic features most associated with the risk of recurrence are wavelet-LLH_glcm_Correlation, wavelet-LLH_glcm_DifferenceEntropy, wavelet-LLH_gldm_DependenceNonUniformity, respectively. Wavelet-LLH_glcm_Correlation quantifies how correlated a pixel is to its neighbor in the LLH wavelet space. High values indicate organized tissue structures, e.g., regular tumor stroma, and low values suggest chaotic tissue patterns. Wavelet-LLH_glcm_DifferenceEntropy calculates the entropy of gray-level difference distribution. Higher values indicate more heterogeneous tissue patterns. Wavelet-LLH_gldm_DependenceNonUniformity measures the variability of gray-level dependencies in local neighborhoods. It reflects complex microenvironment interactions in the CRLM tumor.

The radiomics features extracted by ROI_Intra+Peri4mm_ are shown in **Table 5**. The top 3 radiomic features most associated with the risk of recurrence are wavelet-HHL_glszm_HighGrayLevelZoneEmphasis,wavelet-HHL_glszm_LargeAreaEmphasis, wavelet-HHL_glszm_ZoneEntropy, respectively. Wavelet-HHL_glszm_HighGrayLevel ZoneEmphasis measures the relative distribution of high gray-level zones and emphasizes zones with higher gray-level values within the HHL wavelet-transformed image space. It may correlate with regions of active tumor metabolism or hypervascularized subregions. Wavelet-HHL_glszm_LargeAreaEmphasis quantifies the distribution of large homogeneous zones and emphasizes larger zone sizes within the HHL wavelet space. It may relate to areas of tumor stability or defined growth patterns. Wavelet-HHL_glszm_ZoneEntropy assesses the randomness or disorder in the distribution of zone sizes throughout the image, specifically within the HHL wavelet-transformed space. High values suggest a more irregular and heterogeneous distribution of tumor zones, indicating complex microenvironmental interactions or varied cellular compositions. Low values imply a more uniform and organized arrangement of tumor zones.

The radiomics features extracted by ROI_Intra+Peri6mm_ are shown in **Table 6**. The top 3 radiomic features most associated with the risk of recurrence are wavelet-LLL_glrlm_GrayLevelNonUniformityNormalized, wavelet-LLL_glrlm_LongRunEmphasis, log-sigma-5-mm-3D_glcm_JointEntropy, respectively. Wavelet-LLL_glrlm_GrayLevelNonUniformityNormalized quantifies gray-level distribution uniformity within runs. It may be related to infiltrative growth patterns with irregular cellular density. Wavelet-LLL_glrlm_LongRunEmphasis detects large-scale and spatially coherent tissue regions in LLL-filtered images. Higher values may correlate with well-organized tumor architectures. Log-sigma-5-mm-3D_glcm_JointEntropy enhances edges at 5 mm resolution and captures mid-range heterogeneity.

The inclusion of peritumoral regions (Peri_2mm_, Peri_4mm_, Peri_6mm_) consistently improved model performance compared to intralesional features alone (ROI_Intra_). For example, in the training set, MLP’s AUC increased from 0.872 (ROI_Intra_) to 0.929 (ROI_Intra+Peri4mm_), while in the test set, the best-performing model (MLP with ROI_Intra+Peri4mm_) achieved an AUC of 0.855, compared to 0.735 for SVM with ROI_Intra_ alone. The performance of recurrence risk prediction models improved significantly with the inclusion of peritumoral radiomics features, confirming that tumor-stromal interactions in the peritumoral microenvironment contribute to metastatic progression. Among the evaluated radiomic regions - ROI_Intra_, ROI_Intra+Peri2mm_, ROI_Intra+Peri4mm_, and ROI_Intra+Peri6mm_ demonstrated the best balance between sensitivity and specificity across all machine learning models. Although the SVM model demonstrated a marginal improvement in AUC (+0.002) when expanding the peritumoral region from ROI_Intra+Peri4mm_ to ROI_Intra+Peri6mm_ in the test set, both the RF and MLP models exhibited superior performance with the smaller ROI_Intra+Peri4mm_ signature. This suggests that, for these algorithms, the inclusion of excessive peritumoral information may introduce noise or redundancy, thereby diminishing predictive accuracy. The optimal peritumoral expansion size appears to be context-dependent, with smaller regions (4 mm) potentially striking a better balance between feature informativeness and model generalizability. In contrast, the minimal peritumoral inclusion (ROI_Intra+Peri2mm_) failed to capture sufficient prognostic information, underscoring the importance of an optimized radiomic capture radius. , 

Machine learning model selection significantly influenced predictive accuracy. The selection of SVM, RF, and MLP was based on their distinct strengths and wide applicability in radiomic research. SVM was used for its robustness in handling high-dimensional data and its ability to find optimal hyperplanes for classification, which is valuable given the high dimensionality of our radiomic feature set. RF was selected due to its superiority in capturing non-linear relationships and interactions between features, as well as its built-in feature importance evaluation. MLP was included to account for potential complex, non-linear patterns in the data that traditional statistical models might miss, leveraging its capability to model hierarchical feature representations. The performance of SVM, RF, and MLP models varied significantly across different radiomics signatures, both in the training and test sets. MLP demonstrated superior performance in both training and testing, particularly when incorporating peritumoral features, likely due to its ability to model hierarchical feature interactions. RF showed consistent performance across signatures but generally lagged behind MLP, suggesting that ensemble methods may not fully exploit the radiomics feature space in this context. SVM, while computationally efficient, exhibited the greatest performance decline in testing (e.g., AUC dropped from 0.893 to 0.806 for ROI_Intra+Peri4mm_), highlighting its vulnerability to overfitting in high-dimensional radiomics data. The superior performance of the MLP can be attributed to its unique ability to capture the complex, non-linear relationships and hierarchical feature interactions inherent in our radiomic dataset, particularly the combined intratumoral and peritumoral features. Unlike SVM, which focuses on finding optimal hyperplanes for binary classification, or RF, which relies on ensemble decision trees, MLP’s multi-layered neural network structure allows it to model intricate patterns across high-dimensional radiomic features. In our dataset, recurrence risk is influenced by a confluence of factors, including tumor size, margin status, and peritumoral inflammatory changes, features that manifest as non-linear associations in imaging data. Additionally, the MLP’s capacity for incremental learning allowed it to adapt to the subtle variations in imaging protocols between our training and external validation cohorts, contributing to its stable performance across both datasets.

In real-world clinical settings, for patients newly diagnosed with CRLM, the model can predict the risk of postoperative recurrence before surgery. For instance, patients at high recurrence risk may require more aggressive treatment strategies, such as expanding the scope of liver resection, combining with radiofrequency ablation, or administering preoperative neoadjuvant chemotherapy to reduce the activity of micrometastases. In contrast, patients at low risk can adopt more conservative surgical approaches, such as local hepatic segment resection, to avoid the risk of complications caused by overtreatment. Postoperative follow-up is crucial for reducing the mortality rate of recurrent CRLM. This study can assist physicians in formulating differentiated follow-up plans for patients with different risk stratifications. For example, patients at high risk need shorter follow-up intervals and should be prioritized for more sensitive monitoring methods, patients at low risk can have extended follow-up cycles, which reduces unnecessary medical interventions and the economic burden on patients while optimizing the allocation of medical resources.

To promote the transformation of the model from the research stage to a routine clinical tool, the following steps need to be completed in phases: 1) The current model is built based on data from two centers. The next step will involve conducting external validation in centers across different regions and with varying levels of medical resources to verify the stability of the model under different CT equipment parameters. For any biases identified during validation, the model’s adaptability can be optimized through standardized image preprocessing or transfer learning algorithms. 2) The model needs to be embedded into the hospital’s existing information systems to realize an automated process of image upload, feature extraction, and risk score generation. 3) Multidisciplinary training is required to help medical staff understand the model’s principles, scope of application, and limitations, thereby avoiding over-reliance on the model or misjudgment of risks. 4) To promote the model as a clinical decision-making tool, it is necessary to complete the application process in accordance with medical device regulatory requirements and provide data on its efficacy and safety. The goal is to gradually transition the model from a research tool to a routine clinical auxiliary method, ultimately supporting the individualized management of CRLM patients and improving their prognosis.

Our study has several limitations that warrant consideration. First, the retrospective design inherently introduces potential biases, such as selection bias in patient enrollment and variability in clinical data collection across different time periods. These factors may constrain the generalizability of the model’s predictive performance, as the retrospective setting cannot fully simulate real-world clinical scenarios where patient management is dynamic and influenced by evolving clinical practices. Second, while we performed external validation to test the model’s robustness, the sample size of the external cohort was relatively small, which limits the statistical power to detect subtle differences in predictive accuracy. A larger external validation cohort, ideally from multiple centers with diverse patient populations and clinical practices, would be necessary to confirm the model’s scalability and adaptability across different healthcare settings. Additionally, the absence of molecular genetic data such as KRAS or BRAF mutation in the dataset precluded deeper exploration of the biological underpinnings of recurrence risk, thereby restricting the study’s translational impact. To address these limitations, we plan to conduct prospective, multi-center studies with larger sample sizes in the future. These studies will standardize data collection protocols to minimize bias, and incorporate comprehensive molecular profiling to bridge radiomic features with their biological bases. Expanding the external validation cohort to include more diverse patient populations will further enhance the model’s clinical applicability. Through these efforts, we aim to refine the model and strengthen its potential for translation into clinical practice.

## Conclusion

In conclusion, MLP - based models using ROI_Intra+Peri4mm_ radiomics signatures may offer the best trade-off between predictive accuracy and generalization for recurrence risk stratification in CRLM. This study is expected to have a positive impact on the level of personalized diagnosis and treatment for CRLM patients, as well as on the accuracy of predicting recurrence risk, ultimately enhancing patients’ survival benefits.

## Data Availability

The raw data supporting the conclusions of this article will be made available by the authors, without undue reservation.
